# Inhibitory effects of tamoxifen and doxorubicin, alone and in combination, on the proliferation of the MG63 human osteosarcoma cell line

**DOI:** 10.3892/ol.2013.1487

**Published:** 2013-07-24

**Authors:** ZHENG-XIAO OUYANG, XIAN-AN LI

**Affiliations:** Department of Orthopaedics, Hunan Provincial Tumor Hospital and Tumor Hospital of Xiangya School of Medicine, Central South University, Changsha, Hunan 410013, P.R. China

**Keywords:** osteosarcoma, estrogen receptor, tamoxifen, doxorubicin

## Abstract

The present study aimed to compare the combined effect of tamoxifen (TAM) and doxorubicin (ADM) with the individual effects of TAM and ADM alone on the MG63 human osteosarcoma cell line. Estrogen receptor (ER) expression was detected in the MG63 cells using reverse transcription PCR. The morphological changes during the inhibition of cell growth were observed using an inverted microscope and a 3-(4, 5-dimethy1-2-thiazol-2-yl)-2,5-diphenyl-tetrazolium bromide (MTT) colorimetric assay following the individual or combined addition of TAM and ADM. ERα and ERβ expression was detected in the MG63 cells. The typical apoptotic cell morphology was observed in all groups, with the exception of the control group. The MTT colorimetric analysis demonstrated that the rate of inhibition of cell proliferation in the combination group was significantly increased compared with that in the other groups (P<0.05). ERα and ERβ expression was detected in the MG63 human osteosarcoma cells. TAM and ADM alone were able to inhibit cell proliferation. The combination of TAM and ADM significantly enhanced the inhibitory effect, partly through the enhanced sensitivity of the cells to ADM by TAM, which caused the inhibition of cell proliferation and apoptosis.

## Introduction

Osteosarcoma is the most common primary tumor of the bone. Patients with the condition had a poor prognosis until the 1970s. Major progress was made with the introduction of neoadjuvant chemotherapy, which combines doxorubicin (ADM), cisplatin, methotrexate and/or ifosfamide, resulting in a 5-year survival rate of 60–70% (1–4). However, the rates have not improved further (5). Certain patients, including 74% with lung metastases at the time of diagnosis, have a poor response to neoadjuvant chemotherapy (6), in which treatment-related toxicity and mortality are the main limiting factors allowing no further treatment intensification. Furthermore, a higher dosage of chemotherapy has been shown to result in more long-term complications, including cardiac failure (7,8). Human osteoskeletal non-neoplastic and neoplastic disorders are diseases in which gender differences are markedly noted in the incidence and development. The highest incidence of osteosarcoma is observed in a younger patient group with high levels of sex hormone and sex steroid receptor activity. Estrogen receptors (ERs) have been sporadically reported in human osteosarcoma or its cell lines. Sex steroids and receptors play significant roles in the regulation of cell proliferation in human osteosarcoma.

Tamoxifen (TAM), a selective ER modulator (SERM), has been used widely as the first-line drug of breast cancer chemotherapy with little side effects (9). Studies have shown that TAM may have the ability to enhance the relative sensitivity of tumors of non-sex-hormone-targeted organs to chemotherapeutics (10). As TAM and ADM have antitumoral properties, a combination of these medications may be an option in the therapy of osteosarcoma. However, no data regarding their potential synergistic effects are currently available. Therefore, the present study examined ER expression in the MG63 human osteosarcoma cell line and compared the combined effect of TAM and ADM with the individual effects of TAM and ADM in MG63 human osteosarcoma cells.

## Materials and methods

### Cells and cell culture

The MG63 human osteosarcoma cell line was provided by the Chinese Academy of Science (Shanghai, China). The cells were cultured in Dulbecco’s modified Eagle’s medium (DMEM), supplemented with 10% fetal calf serum and antibiotics, at 37°C in a humidified atmosphere with 5% CO_2_. All cell culture reagents were purchased from Gibco Co., Ltd. (Carlsbad, CA, USA).

### Analysis of ER mRNA expression

Due to the low expression of ER in osteoskeletal cells (11) and the difficulty in examining ER through immunohistochemistry, reverse transcription (RT) PCR for ERα and ERβ was performed on the MG63 cells. Total RNA was extracted using TRIzol (Invitrogen Life Technologies, Carlsbad, CA, USA) from the MG63 cells according to the manufacturer’s instructions. First-strand cDNA was synthesized from 1 μg total RNA by reverse transcription using oligo-dT primers and reverse transcriptase (Invitrogen Life Technologies) according to the manufacturer’s instructions. cDNA was utilized as a template for the amplification of full-length ERα and ERβ using the following primers: 5′-TTGCTATGTTACTAAGCGTGAG-3′ for ERα and 5′-GATGCTTTGGTTTGGGTGATT-3′ for ERβ. PCR was performed in 50 μl volumes containing 1 μl cDNA, 1 μl (10 μM) of each primer and 4 μl (2.5 mM) of each dNTP in a reaction buffer containing 1 μl (2.5 u/μl) Taqplus (Invitrogen Life Technologies). The thermocycling conditions consisted of an initial incubation step at 95°C to activate the polymerase enzyme, followed by 35 cycles consisting of 45 sec at 95°C, 45 sec at 60°C and 45 sec at 72°C. The PCR products were separated in 2% agarose gels and visualized by ethidium bromide staining. The housekeeping gene, β-actin (5′-AGGGGCCGGACTCGTCATACT-3′), was used as a control.

### Drugs and drug treatment

The MG63 cells were divided into three groups according to the incubation time (24, 48 and 72 h). Each group was treated with various concentrations of TAM (First Pharmaceuticals, Hangzhou, China) and ADM (Farmitalia Carlo Erba, Milan, Italy). According to the clinical serum concentrations of the two drugs, concentrations of 0.1, 1, 5 or 10 μg/ml ADM and 1, 2, 5 or 10 μmol/l TAM were used for the individual groups, while 5 μg/ml ADM and 5 μmol/l TAM were used for the AT (ADM and TAM) combination group.

### Morphological changes of cell growth inhibition

The MG63 osteosarcoma cells were cultured to the exponential phase of growth and the cell number was adjusted to 1.0×10^5^ cells/ml and seeded in 25-cm^3^ tissue culture flasks. Subsequent to the cells being incubated for 24 h, the medium was replaced by a culture medium that contained the various concentrations of drugs, and the cells were incubated for another 24, 48 and 72 h. The morphological changes during cell growth inhibition were observed using an inverted microscope.

### 3-(4,5-Dimethy1-2-thiazol-2-yl)-2, 5-diphenyl-etrazolium bromide (MTT) activity measurement

The MTT assay is a colorimetric measurement of MTT reduction to a blue formazan product by mitochondrial dehydrogenases of viable cells (12). The cells were seeded in 96-well plates at a density of 5×10^3^ cells/well and incubated for 24 h. Thereafter, the medium was replaced and incubation continued for a further 24, 48 and 72 h in the presence or absence of the test compounds of the various concentrations of the drugs. The medium was replaced and 50 μl MTT (Sigma Chemical Co., St. Louis, MO, USA) working solution was added to each well at a final concentration of 0.5 mg/ml. The cells were incubated for 4 h at 37°C and the medium was changed to 100 μl dimethyl sulfoxide to dissolve the formazan. Optical density (A) was measured at 492 nm of the wave length using a Tecan Spectrafluor Plus microplate spectrophotometer (Esbe Scientific Industries Inc., Ontario, Canada). Each experiment was performed in triplicate. The medium of the control group was replaced by DMEM supplemented with 10% fetal calf serum and antibiotics. The quantity of viable cells was reflected by the cell growth inhibition rate that was calculated using the following formula: Cell growth inhibition rate (%) = (1 − optimum A of experimental group/optimum A of control) × 100.

### Statistical analysis

The results are expressed as the mean ± standard error. The data were evaluated by a multivariate analysis of single variance with repeated measures designs. Multiple comparisons were performed using one way analysis of variance (ANOVA) by a Student-Newman-Keuls test. For each variable, at least three independent experiments were performed. All analyses were performed using SPSS 20.0 software (SPSS, Inc., Chicago, IL, USA). P<0.05 was considered to indicate a statistically significant difference.

## Results

### ER expression in MG63 cells

**Figure 1:**
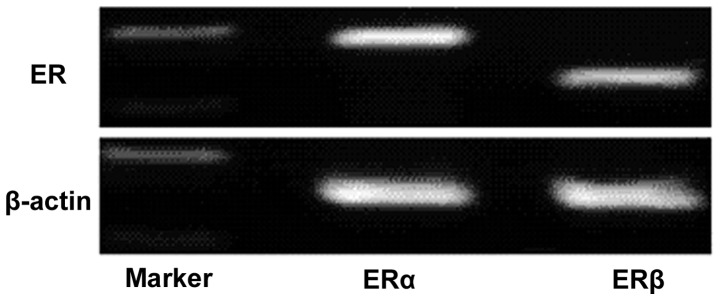
RT-PCR analysis of ER (top) and β-actin (bottom) in MG63 cells. RT, reverse transcription; ER, estrogen receptor.

RT-PCR analysis of mRNA that was isolated from the MG63 human osteosarcoma cell line indicated the presence of ERα and ERβ mRNA in the MG63 cells, suggesting that ERα and ERβ are constitutively expressed by the MG63 human osteosarcoma cell lines (Fig. 1).

### Morphological analysis of MG63 cells with various drug intervention

**Figure 2:**
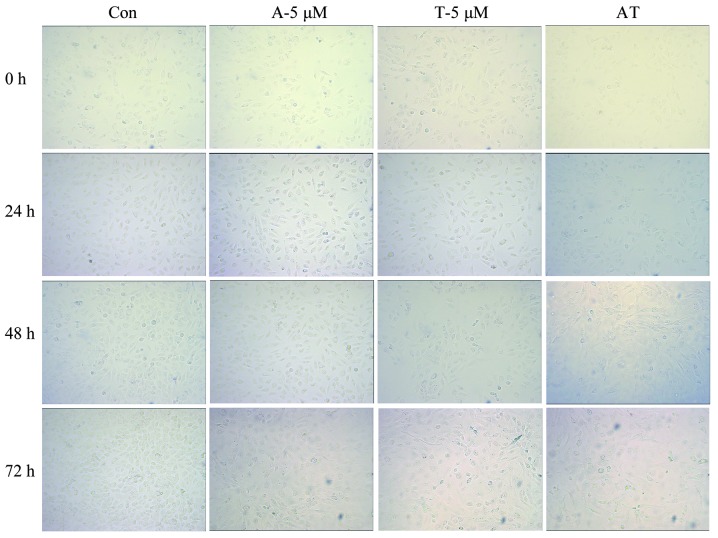
Morphological analysis of MG63 cells with various interventions of drugs. Con, control; A, ADM (doxorubicin); T, TAM (tamoxifen); AT, ADM and TAM.

Typical apoptotic cell morphology was observed in all the groups, with the exception of the control group. The images show that the cells grew well in the control group, and were mostly spindle-shaped and large, with uniform round nuclei and clear cytoplasm. It was identified that 24 h following the administration of TAM (5 μmol/l), a small number of cells became round-shaped and multiplied slowly. With a prolonged amount of time and an increased drug concentration, more cells became round-shaped and dark. Additionally, the number of fragments increased and the sizes and amount of the plasma granules within the cells changed. Cell shrinkage and the formation of apoptotic bodies were observed. In the ADM group (5 μg/ml, 24 h), the typical early stage of cell apoptosis changes, including cell membrane budding and swollen organelles, were observed. The same morphological changes as previously described were observed in the combination group (AT) and the rate of apoptosis in the combination group was higher than that in the single drug groups under the same treatment times (Fig. 2).

### MTT colorimetric analysis

It is well-known that the MTT reagent directly reacts with the mitochondria (mitochondrial dehydrogenase) of metabolically active cells. Therefore, the reaction of MTT reduction is directly proportional to the number of growing cells. The measured optical density is directly proportional to the number of viable cells in the culture medium. Therefore, MTT is regarded as a quantitative assay to determine the cytotoxicity of the materials and the viability/proliferation of the cells in solution in various groups.

**Figure 3:**
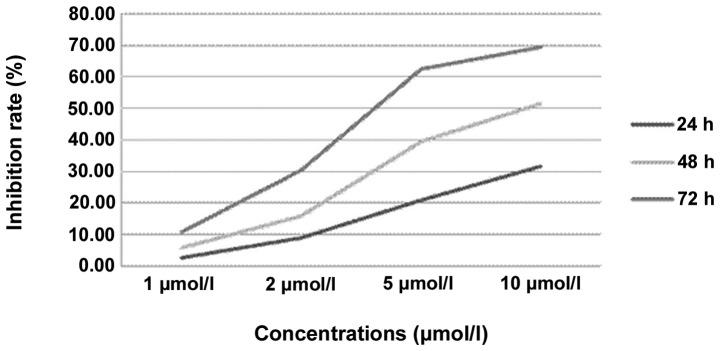
Effect of various concentrations and treatment time of TAM on MG63 cells. TAM, tamoxifen.

Table I and Fig. 3 show the effect of TAM on the cell growth inhibition rate. According to the statistical analysis, the cell growth inhibition rates in the TAM groups gradually increased with a prolonged treatment time and increased drug concentrations. A significant difference was observed between the various groups with various times and concentrations. However, in the multiple comparisons, there were no significant differences between the 24- and 48-h, 1- and 2-μmol/l or 5- and 10-μmol/l groups (P>0.05). There were obvious dose-effect and time-effect associations in the MG63 cells that were treated with TAM.

**Figure 4:**
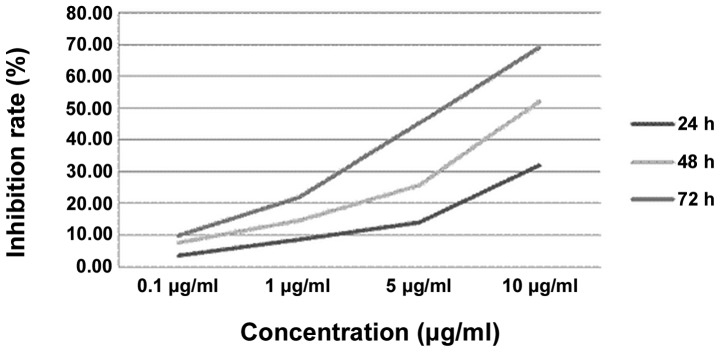
Effect of various concentrations and treatment time of ADM on MG63 cells. ADM, doxorubicin.

Table II and Fig. 4 show the effect of ADM on the cell growth inhibition rate. The statistical analysis shows that the cell growth inhibition rates in the ADM groups gradually increased with a prolonged treatment time and increased drug concentrations. A significant difference was observed between the groups with the various times and concentrations. However, in the multiple comparisons, there were no significant differences between the 24- and 48-h, 72- and 48-h or 0.1- and 1-μg/ml groups (P>0.05). There were obvious dose-effect and time-effect associations in the MG63 cells that were treated with ADM.

**Figure 5:**
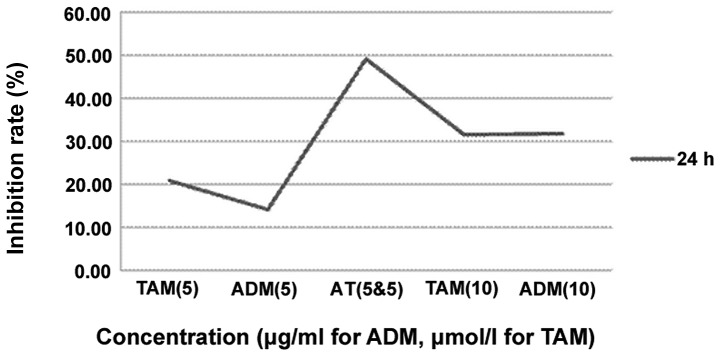
Effect of various concentrations and combination of drugs on MG63 cells. TAM, tamoxifen; ADM, doxorubicin; AT, ADM and TAM.

Table III and Fig. 5 show the effects of TAM (5 and 10 μmol/l, respectively), ADM (5 and 10 μg/ml, respectively) and AT (combination of 5 μmol/l TAM and 5 μg/ml ADM) on the cell growth inhibition rate. By the statistical analysis, the cell growth inhibition rates in the ADM groups gradually increased with a prolonged treatment time. A significant difference was observed between the group with various times and concentrations. However, in the multiple comparisons, there were no significant differences between the TAM (5 μmol/l) and ADM (5 μg/ml) groups, TAM (5 μmol/l) and TAM (10 μmol/l) groups, TAM (5 μmol/l) and ADM (10 μg/ml) groups and the TAM (10 μmol/l) and ADM (10 μg/ml) groups (P>0.05). The inhibition rate of the combination group was higher than that of the single drug groups, including the single drug groups with double the concentration.

## Discussion

Non-neoplastic bone diseases, including osteoporosis and osteoarthritis, have a higher incidence in females, and osteoporosis may be treated and prevented using 17β-estradiol and raloxifene to a certain degree. Estrogen has been shown to play a significant role in the proliferation of bone cells (13). Furthermore, the highest incidence of osteosarcoma is observed in a younger patient group with a high level of sex hormone activity. Sex steroids and receptors play significant roles in the regulation of cell proliferation in human osteosarcoma.

Walker *et al* first reported the existence of sex steroid receptors in four cases of osteosarcoma from a dextran-coated charcoal assay (14). Stedman *et al* also identified ER proteins in osteosarcoma from gel filtration in the same year (15). These were the first studies to demonstrate the potential correlation between sex steroids and osteosarcoma. More recent studies on ER expression in osteosarcoma cases and cell lines are rare. The majority of the studies found that ERα and ERβ were expressed in human osteoblasts. The analysis of the ER subtypes reported by Chen *et al* demonstrated the dominance of ERβ in the MG63 cells (16). Dohi *et al*(17) reported that ERβ was relatively widely distributed in the osteosarcoma cases and ERβ was the predominant ER that was expressed in the MG63 cells. In other osteosarcoma cell lines, including U2OS, ERα and ERβ mRNA has also been reported to be detected at a ratio of 1:4. (18) Solakidi *et al* reported that ERα was localized mainly in the nucleus of human U2OS osteosarcoma cells and ERβ was specifically enriched at the site of the mitochondria, but its significance has remained unknown at this juncture (19). The results of the present study revealed expression of ERα and ERβ mRNA in the MG63 cells, as detected by RT-PCR, which was consistent with those that have been reported previously. Therefore, the present study provides a basis for the endocrine therapy of osteosarcoma.

Furthermore, Dohi *et al*(17) reported that the proliferation of MG63 human osteosarcoma cells was stimulated by E2 and that the increment are significantly suppressed clinically using well-established blockers of a corresponding steroid that had been previously reported by Luo and Liao (20). These findings indicated the potential role of the ER in the pathogenesis and development of osteosarcoma. The steroid blockers have the potential to be used as suppressors of cell proliferation of human osteosarcoma cells. Therefore, estrogen is considered to exert effects not only on non-neoplastic bones but also on their neoplasms, particularly osteosarcomas.

As an ER antagonist, TAM is extensively used in the treatment of mammary adenocarcinoma. The most common side effect is vasomotor symptoms, which have been reported in 1–4% of cases. Toxicity and other side effects have been relatively uncommon during the clinical use of the TAM. TAM has been demonstrated to have biological and pharmacological activities beyond its traditional role as an anti-estrogenic agent. Among these are the inhibition of multidrug resistance (MDR) (21–23), protein kinase C (PKC) (24), calmodulin (25), insulin growth factor (26) and transforming growth factor-α (27). In addition, TAM has been associated with transforming growth factor-β1 induction (28), immune reaction modulation (29), apoptosis induction (30,31) and a reduction in the fluidity of the cytoplasmic membrane (32). It has been speculated that certain activities may be responsible for the unexpected therapeutic effect of TAM, alone or in combination with other anti-cancer drugs, in various cancers, including malignant melanoma (33,34), brain glioma (35) and lymphoma (36). In the present study, there were obvious dose-effect and time-effect associations in the MG63 cells that were treated with TAM. The therapeutic effect of TAM alone may be mediated by its non-specific effect on cytoplasmic membranes, as TAM, a triphenylethylene, is lipophilic and is expected to partition into hydrophobic domains in the fluid mosaic structure of cell membranes. Clark *et al*(32) have demonstrated previously that TAM, at concentrations of >1 mM, significantly decreased the fluidity of the plasma membrane of ER^−^ breast cancer cells and may have contributed to its non-ER-mediated cytotoxicity.

The analysis of sex steroid receptors in resected specimens of osteosarcoma cases and cell lines as a potential surrogate marker may be required in order to prepare for a potential endocrine therapy, particularly in the instance that the patients develop pulmonary metastasis and a poor response to the chemotherapy in their clinical course. Ferguson *et al*(37) reported that TAM showed the ability to enhance the survival rate of advanced breast cancer patients who had been treated with ADM in phase III. Studies have also indicated that the drug resistance of neuroblastoma, small cell lung cancer, gastric cancer, liver cancer, bladder cancer, ovarian cancer and epirubicin may all be reversed using TAM (38). The potential synergistic cytotoxic effect between TAM and chemotherapeutic agents in estrogen-independent solid tumors has been studied and documented (31,39).

In an effort to increase the clinical efficacy of ADM, the present study determined whether the antitumor effects of this drug were able to be enhanced using a concurrent application of other drugs that have demonstrated anti-osteosarcoma activity, such as TAM. In the present study, the inhibition rates of the 24-h group of the MG63 human osteosarcoma cell line by ADM (5 μg/ml), TAM (5 μmol/l), ADM (10 μg/ml) and TAM (10 μmol/l) were 14.04, 20.85, 31.78 and 31.52%, respectively. By contrast, following the combination of TAM and ADM, the inhibitive rates increased to 49.17% and the results were consistent with those of the other groups, indicating that the effect of the chemotherapy drugs was enhanced by the cooperation of the two drugs and that TAM may enhance the effect of chemotherapeutics by comparing ADM and ADM mixed with TAM. The present analyses demonstrated that combination treatment of MG63 osteosarcoma cells exerts greater antiproliferative and apoptosis-stimulatory effects than treatment with the individual drugs alone.

Cancer cells may gradually develop a resistance to chemotherapeutic drugs in the course of chemotherapy and there have been numerous studies with regard to overcoming the resistance mechanisms (40). TAM, a cholangiocarcinoma sensitizer, may act as a P-glycoprotein (gp) substrate by competing for the P-gp binding site with antineoplastic agents. TAM inhibits the function of the transmembrane transporter and lowers the velocity of drug efflux from within cells, leading to an enhancement of the drug concentration and the effect of the chemotherapeutic drugs. Another underlying molecular event affecting this enhancement process appears to be the potent downregulation of Bcl-2, a critical anti-apoptotic and chemoprotective protein. It has been well established that the ratio of the intracellular amount of the two proteins, Bcl-2 (antiapoptotic) and Bax (proapoptotic), is critical for subsequent cell survival and cell death (41,42). Bax is the major apoptosis-promoting gene and may explain the synergistic cytotoxicity of TAM with ADM in the treatment of osteosarcoma. TAM also exerts a pleiotropic effect on intracellular growth and survival pathways, in particular the inhibition of PKC (43,44), which is a growth-stimulatory kinase. Cheng *et al*(45) reported that high-dose TAM may potentiate ADM-induced apoptosis of hepatocellular carcinoma cells and this effect of TAM is associated with its inhibition on the membrane translocation of PKC-α and is not mediated by MDR inhibition. Biochemical modulation as a measure to improve systemic chemotherapy for osteosarcoma requires further investigation. Appropriate treatment strategies that are formulated according to the mechanisms of drug resistance and synergistic cytotoxic effects between TAM and ADM may be guides in the fight against cancer and thus require investigation. Due to the low prevalence rate of osteosarcoma, breakthroughs in new therapeutic approaches based on gene technology may be difficult.

To the best of our knowledge, no studies have been reported that are concerned with the use endocrine therapy for treating patients with osteosarcoma and that include an analysis of the synergistic cytotoxic effect on osteosarcoma cell lines between TAM and ADM. However, these studies should be pursued, particularly the molecular mechanism that is complicated by the fact that TAM is able to exert independent antitumor effects, considering the relatively poor clinical outcome of osteosarcoma patients with pulmonary metastases and a poor response to chemotherapy.

In conclusion, ADM, combined with TAM, is able to enhance the killing of tumor cells, and the addition of TAM may be a beneficial adjuvant to ADM-based chemotherapy in the treatment of osteosarcoma. Therefore, it may be worthwhile to consider this combination regimen for further evaluation in clinical trials. This information may be used to improve osteosarcoma treatment by reversing or reducing drug resistance, therefore supporting the notion that these drug combinations should be further evaluated in clinical trials.

## Figures and Tables

**Table I:** Cell growth inhibition rate of various concentrations of TAM (%).

	Concentrations of TAM (μmol/l)				
	
Time (h)	0	1	2	5	10
24	0	2.49±0.21	8.80±0.56	20.85±1.15	31.52±1.54
48	0	5.68±0.32	15.67±0.62	39.57±0.61	51.56±1.85
72	0	10.64±0.44	30.38±0.76	62.51±1.24	69.40±2.24

TAM, tamoxifen.

**Table II:** Cell growth inhibition rate of various concentrations of ADM (%).

	Concentrations of ADM (μg/ml)				
	
Time (h)	0	0.1	1	5	10
24	0	3.63±0.16	8.75±0.93	14.04±1.47	31.78±1.68
48	0	7.65±0.41	14.46±1.56	25.53±2.07	51.99±2.03
72	0	9.89±0.84	21.76±1.89	45.23±2.64	68.95±3.65

ADM, doxorubicin.

**Table III:** Cell growth inhibition rates of single and combination drugs (%).

	Concentrations (ADM, μg/ml; TAM, μmol/l)					
	
Time (h)	0	TAM (5)	ADM (5)	AT (5 each)	TAM (10)	ADM (10)
24	0	20.85±1.15	14.04±1.47	49.17±3.21	31.52±1.54	31.78±1.68
48	0	39.57±0.61	25.53±2.07	68.33±1.90	51.55±1.83	51.99±2.03
72	0	62.51±1.24	45.23±2.64	85.27±2.15	69.40±2.24	68.95±3.65

TAM, tamoxifen; ADM, doxorubicin; AT, ADM and TAM.
